# Tolerance to paternal genotoxic damage promotes survival during embryo development in zebrafish (*Danio rerio*)

**DOI:** 10.1242/bio.030130

**Published:** 2018-05-15

**Authors:** Cristina Fernández-Díez, Silvia González-Rojo, Marta Lombó, M. Paz Herráez

**Affiliations:** Department of Molecular Biology, Faculty of Biology, Universidad de León, Campus de Vegazana, s/n 24071, León, Spain

**Keywords:** DNA damage response, DNA damage tolerance, DNA repair, Embryo development, Sperm DNA damage

## Abstract

Spermatozoa carry DNA damage that must be repaired by the oocyte machinery upon fertilization. Different strategies could be adopted by different vertebrates to face the paternal genotoxic damage. Mammals have strong sperm selection mechanisms and activate a zygotic DNA damage response (DDR) (including cell cycle arrest, DNA repair and alternative apoptosis) in order to guarantee the genomic conformity of the reduced progeny. However, external fertilizers, with different reproductive strategies, seem to proceed distinctively. Previous results from our group showed a downregulation of apoptotic activity in trout embryos with a defective DNA repairing ability, suggesting that mechanisms of tolerance to damaged DNA could be activated in fish to maintain cell survival and to progress with development. In this work, zebrafish embryos were obtained from control or UV-irradiated sperm (carrying more than 10% of fragmented DNA but still preserving fertilization ability). DNA repair (γH2AX and 53BP1 foci), apoptotic activity, expression of genes related to DDR and malformation rates were analyzed throughout development. Results showed in the progeny from damaged sperm, an enhanced repairing activity at the mid-blastula transition stage that returned to its basal level at later stages, rendering at hatching a very high rate of multimalformed larvae. The study of transcriptional and post-translational activity of *tp53* (ZDF-GENE-990415-270) revealed the activation of an intense DDR in those progenies. However, the downstream pro-apoptotic factor *noxa* (ZDF-GENE-070119-3) showed a significant downregulation, whereas the anti-apoptotic gene *bcl2* (ZDF-GENE-051015-1) was upregulated, triggering a repressive apoptotic scenario in spite of a clear genomic instability. This repression can be explained by the observed upregulation of p53 isoform Δ*113p53*, which is known to enhance *bcl2* transcription. Our results showed that *tp53* is involved in DNA damage tolerance (DDT) pathways, allowing the embryo survival regardless of the paternal DNA damage. DDT could be an evolutionary mechanism in fish: tolerance to unrepaired sperm DNA could introduce new mutations, some of them potentially advantageous to face a changing environment.

## Abstract (precis)

**Summary:** In fish embryos, genomic instability generated by fertilization with DNA damaged sperm activates mechanisms of DNA damage tolerance, which seems to be mediated by Δ113p53 expression, promoting survival.

## INTRODUCTION

Sexual reproduction is a key evolutionary event allowing the combination of genetic information from two progenitors for giving rise to a newborn. In order to assure the correct genomic conformity of the progeny, a highly coordinated series of processes are required, during both gametogenesis and fertilization. The whole process entails specific genomic rearrangements ignoring in some cases the canonical pathways of DNA damage control and repair, thus increasing the chances of introducing mutations.

The male gamete is considered to be the main source of *de novo* mutations produced during fertilization (Crow, 2000; Kong et al., 2012; Marchetti et al., 2015, 2007). Spermatogenesis promotes a number of DNA strand breaks throughout chromosome recombination at meiosis and nuclear condensation. These injuries remain unrepaired in the mature spermatozoa since the post-meiotic spermatids have limited or absent repairing mechanisms and a highly compacted nucleus, which hinders the access to DNA repair machinery (Baarends et al., 2001; Herráez et al., 2015; Olsen et al., 2005). In addition, many genotoxic agents are able to promote different types of DNA lesions post-ejaculation, compromising the sperm genomic stability (reviewed by Herráez et al., 2015). In contrast, DNA repairing activity is maintained during oogenesis. In that case, the mature oocyte contains the mRNAs and proteins in charge of handling a certain level of paternal DNA damage after fertilization (Jaroudi et al., 2009; Jaroudi and Sengupta, 2007; Langley et al., 2014; Marchetti et al., 2007; Ménézo et al., 2010). The genetic conformity of the zygote is the master piece to obtain a healthy progeny. Different works have linked sperm DNA damage with a higher rate of embryo loss as well as defects at birth (González-Marín et al., 2012; Hourcade et al., 2010; Jaroudi and Sengupta, 2007). Moreover, studies in mammals and fish have demonstrated that efficient repair machinery from oocytes is a mandatory condition to allow correct embryo development. In all cases, changes in gene expression related to DNA damage checkpoints and DNA repair deeply affected embryo development (Fernández-Díez et al., 2016, 2015; González-Marín et al., 2012; Jaroudi and Sengupta, 2007; Marchetti et al., 2007). In mammals, fertilization with DNA-damaged sperm (DDS) seems to activate some mechanisms of cell cycle arrest at zygotic G2/M and to increase the activity of different repairing pathways immediately after fertilization (mainly BER, MMR and HR pathways) (Chen et al., 2012; González-Marín et al., 2012; Jaroudi and Sengupta, 2007; Kumar et al., 2013; Wang et al., 2013). Nevertheless, the variable efficiency of those repairing pathways may generate DNA double-strand breaks (DSBs) in the unrepaired spots after DNA duplication. Marchetti et al. (2015, 2007) showed that paternal exposure to ionizing irradiation or mutagenic chemicals promoted chromosomal aberrations affecting sister chromatids (caused by inefficient repair of inter-strand crosslinks) in 64.2% of the zygotes and led to post-implantation death in 45% of the embryos. Studies from the same group also demonstrated in mouse that the genomic aberrations in zygotes promoted by the spermatic damage are highly predictive of abnormal embryonic development (Marchetti et al., 2015). The reproductive outcomes largely depend on the fidelity of the zygotic repair: only those embryos properly repaired or carrying reciprocal translocations after zygotic repair are able to produce viable offspring, whereas the zygotes with failures in DNA repair suffer from genomic aberrations and eventually they may result in preimplantation loss or in dead implants (Marchetti et al., 2004).

Later on, the mammalian preimplantation period is a critical stage in which the transition from maternal to zygotic transcripts takes place (Clift and Schuh, 2014; Li et al., 2013). However, the control of DNA damage still relies on maternal factors (Jaroudi et al., 2009; Jaroudi and Sengupta, 2007). DNA replication and cell proliferation are fast, but the cell cycle is short, increasing the risk of losing genomic integrity and reducing the chances to activate the cycle checkpoints which take over DNA stability (Jaroudi and Sengupta, 2007).

Through development, the embryo acquires a greater ability to generate an integral DDR, activating different genes involved in the detection of DNA damage, in different DNA repairing pathways, in the control of cell cycle arrest and in the apoptotic activity (Jaroudi and Sengupta, 2007). In that way, embryos acquire the capacity to make the appropriate developmental decision after damage: to proceed with the development either tolerating the DNA damage or activating the apoptosis. During mammalian embryo development, all data indicate that the repairing effort starts at one-cell stage, whereas cell death or apoptosis are only observed at late cleavage or blastocyst stages. Therefore, there is a strict control at pre-implantation stages, but a tolerant period during cleavage stages (Jaroudi and Sengupta, 2007; Langley et al., 2014). An increase in apoptotic activity is also observed at organogenesis upon genotoxic stress (Jaroudi and Sengupta, 2007), and a high rate of post-implantation death is observed in embryos with residual unrepaired damage (Marchetti et al., 2004).

Most teleosts, as external fertilizers, display a very different reproductive strategy based on the increased quantity of embryos with low surviving probabilities after birth: large egg batches are fertilized but the embryos, which are exposed to changing environmental conditions, predation, etc, have few chances to survive. Since they have less restrictive mechanisms for sperm selection than mammals, it is likely that fertilization with DDS occurs (Pérez-Cerezales et al., 2010). Moreover, the study of transcripts in trout embryos and larvae suggested a tolerance to genotoxic damage after inhibition of DNA repair in zygotes (Fernández-Díez et al., 2015), as well as in embryos obtained from sub optimal quality oocytes, which showed an altered expression of DDR-related genes (Fernández-Díez et al., 2016). Our hypothesis is that embryo development in fish displays a high degree of tolerance to paternal DNA damage, being able to progress up to hatching, even when embryos carry a high degree of genomic damage. In this study, we use zebrafish as model species in order to analyze the effects of fertilization with DDS on the activation of DDR mechanisms and on the progeny development. Furthermore, zebrafish share with mammals the DNA repairing pathways, cell cycle control mechanisms and the main apoptotic pathways.

## RESULTS

### Genotoxic damage in sperm

**Fig. 1.**
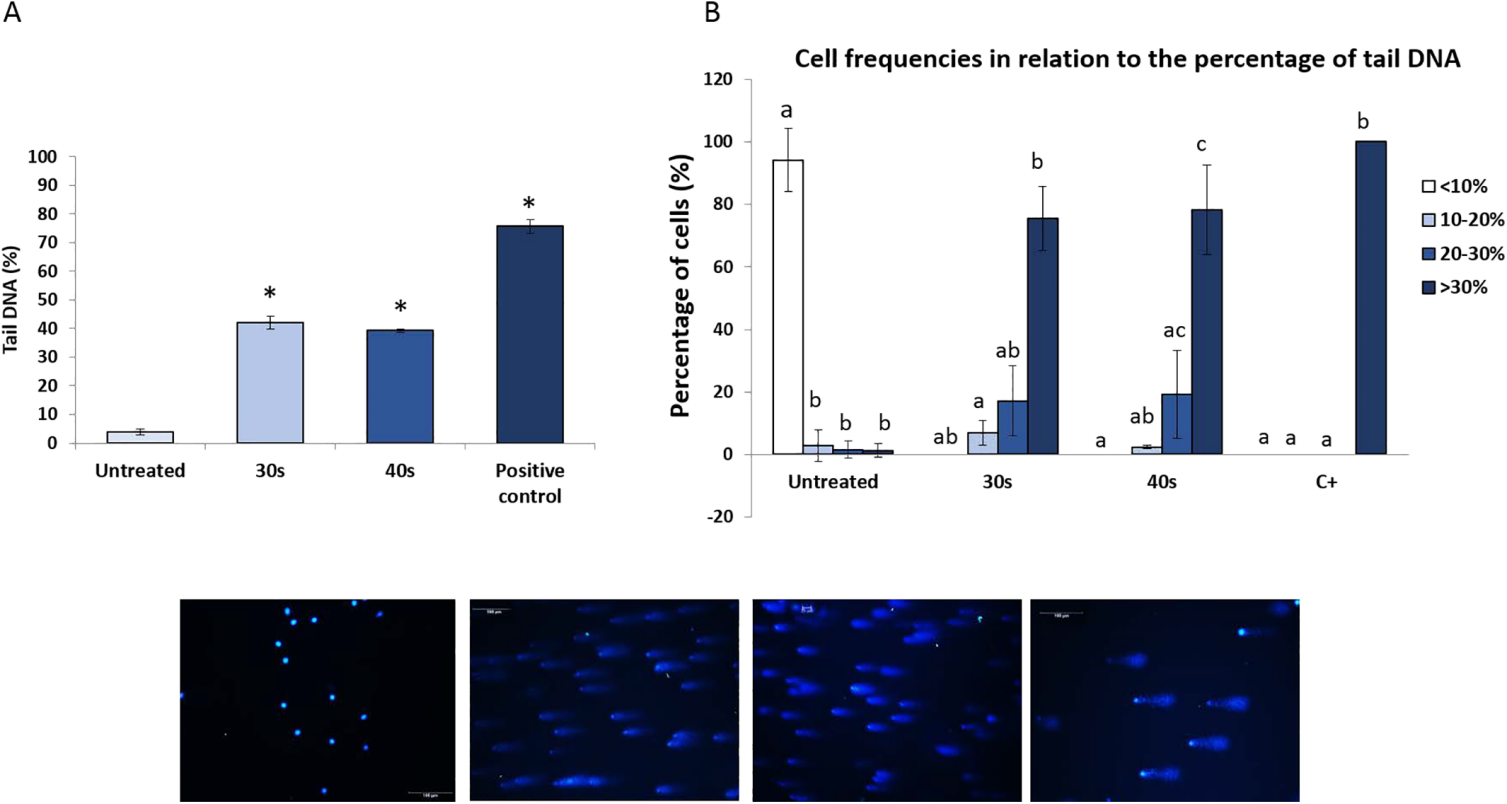
**Sperm DNA integrity.** (A) Percentage of tail DNA by comet assay. Representative comet assay images (20×) from sperm cells are shown (bottom). Scale bars: 100 μm. (B) Four rates of DNA fragmentation are shown for each type of spermatic cells (untreated sperm and sperm treated with 30 s or 40 s of UV irradiation). Data indicate mean±s.e.m. (*n*=3). Different letters and asterisks indicate significant differences (*P*<0.05).

**Fig. 2.**
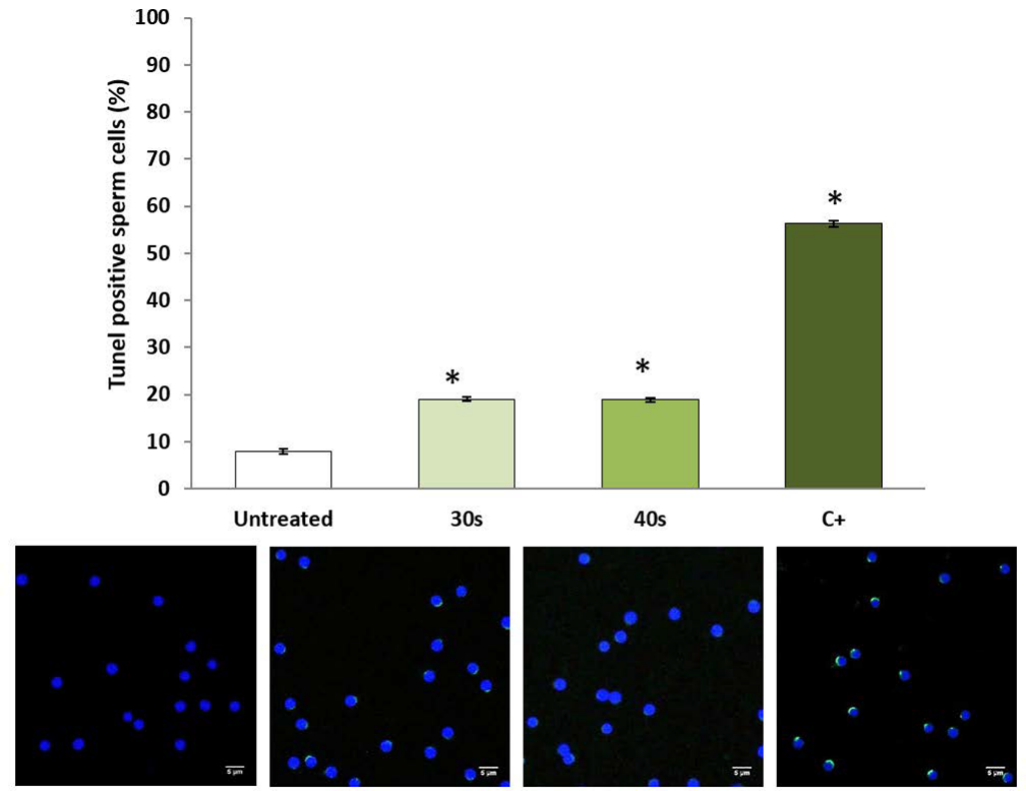
**Relative intensity of TUNEL-positive cells.** Representative TUNEL images (40×) from sperm cells are shown. Scale bars: 5 µm. Data are mean±s.e.m. (*n*=3) (*P<0.05).

The analysis of cells with different degree of DNA damage using the comet assay showed that UV irradiation significantly increased DNA fragmentation. The average percentage of tail DNA, which indicates the percentage of fragmented DNA in cells, increased from 3.84±1.12% in control sperm to 42.09±2.15% and 39.2±0.68% in treated samples (30 s and 40 s of UV irradiation, respectively) (Fig. 1A). In addition, 94.17±5.82% of the control cells contained less than 10% fragmented DNA, whereas in the treated samples there were no cells in that range. Most of them carrying more than 30% fragmented DNA (75.6±5.9% and 78.2±8.22% in samples for irradiated 30 s and 40 s, respectively) (Fig. 1B). TUNEL assay (Fig. 2) also revealed a significant increase in DNA fragmentation, with almost 12% more positive cells in UV-irradiated samples (19±0.38% and 18.89±0.47% in samples irradiated for 30 s and 40 s, respectively) than in control ones (7.91±0.46%).

### Progeny performance

**Fig. 3.**
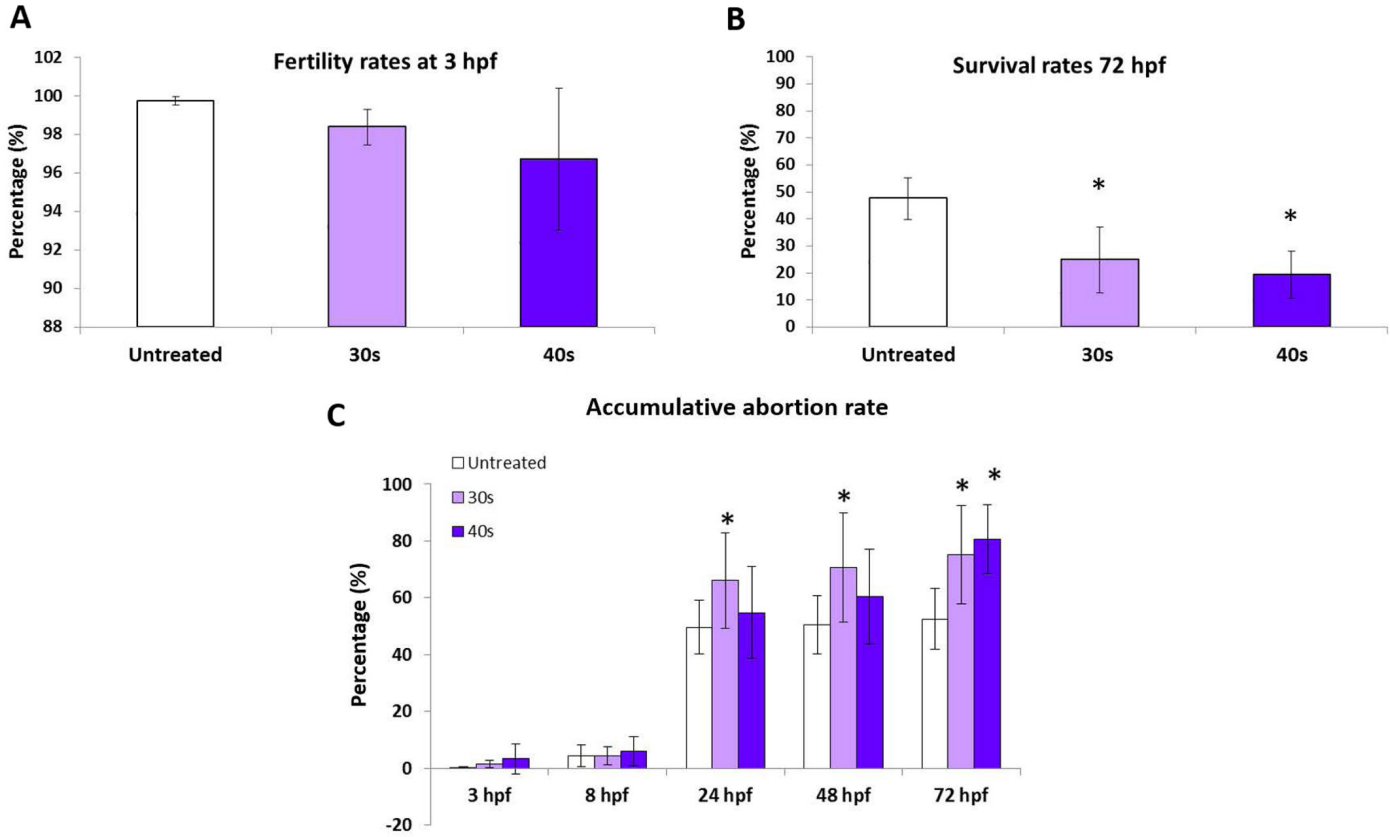
**Progency performance.** (A) Capacity of sperm to fertilize measured at 3 hpf. (B) Survival rates at 72 hpf in progenies obtained from untreated sperm and sperm treated with 30 s and 40 s of UV irradiation. (C) Accumulative abortion rate at 3 hpf, 8 hpf, 24 hpf, 48 hpf and 72 hpf. Data are mean±s.e.m. (*n*=4) (*P<0.05).

As showed in Fig. 3, sperm treatment did not affect the fertility rates (Fig. 3A). The survival rate at 72 hours post fertilization (hpf) was clearly lower in progenies from treated sperm than in those from control ones (Fig. 3B), showing an increase in the mortality rate from 8 hpf to 24 hpf and 72 hpf (Fig. 3C).

**Fig. 4.**
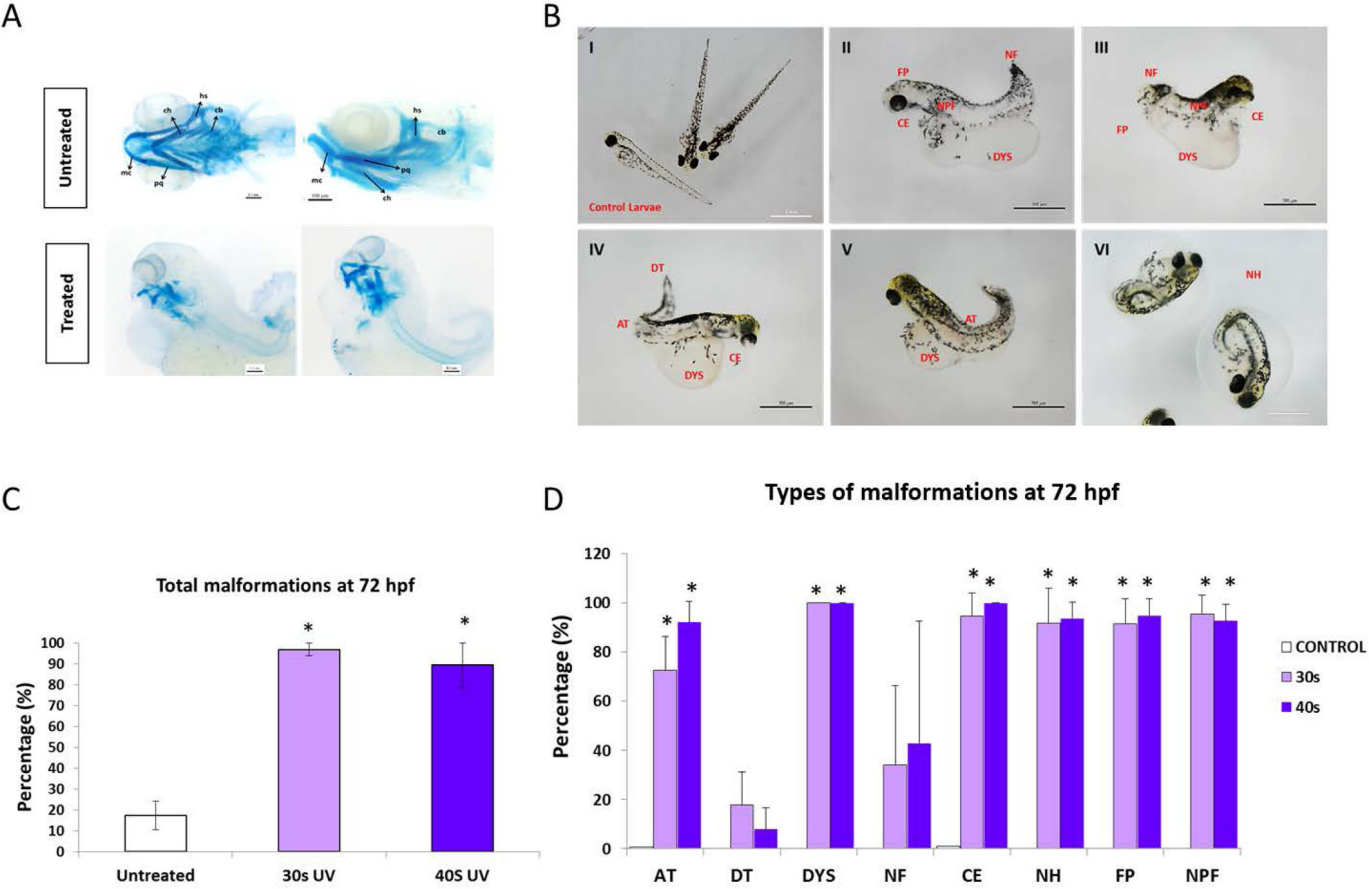
**Phenotypes of larvae from untreated sperm and sperm treated with 30 s and 40 s of UV irradiation.** (A) Representation of mandibular cartilage from zebrafish embryos at 6 dpf. mc, Meckel's cartilage; pq, palatoquadrate; bh, basihyal; ch, ceratohyal; hs, hyosymplectic; cb, ceratobranchial. Scale bars: 100 µm. (B) Types of malformations observed at 72 hpf. AT, axial torsion; DT, distal torsion; DYS, defective yolk sac; NF, no fin; CE, cardiac edema; NH, no hatching; FP, failed pigmentation; NPF, no pectoral fin. Scale bars: 500 µm. (C) Percentage of total malformations. (D) Percentage of specific malformations. Data are mean±s.e.m. (*n*=3) (*P<0.05).

**Fig. 5.**
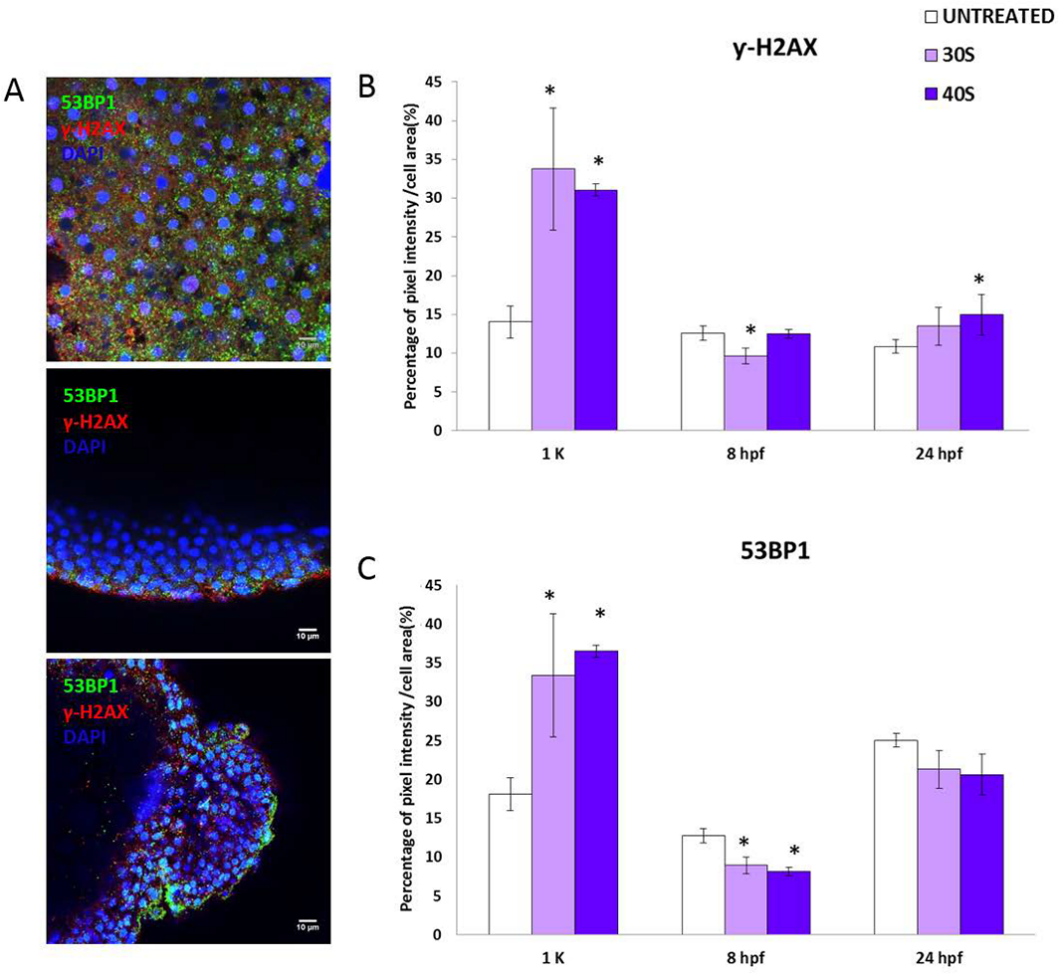
**Repairing ability during embryo development at 1k-cell, 8 hpf, 24 hpf and 72 hpf in progenies from untreated sperm and sperm treated with 30 s and 40 s of UV irradiation.** (A) 53BP1 and γ-H2AX immunolocalization. Representative confocal images (40×) of whole embryos. Cell nuclei were stained with DAPI. Scale bars: 10 µm. (B) Percentage of pixel intensity per cellular area for γ-H2AX. (C) Percentage of pixel intensity per cellular area for 53BP1. Data are mean±s.e.m. (*n*=3) (*P<0.05).

The percentage of malformed larvae at 72 hpf was extremely high in those batches from treated sperm, reaching almost 100% malformation rate (96.87±3.12% and 89.28±10.71% in batches from sperm treated for 30 s and 40 s, respectively) (Fig. 4C,D). Most of the embryos displayed several lesions simultaneously (Fig. 4B), showing a global phenotype modification (axial torsion, cardiac edema, defects in yolk conformation, inability to hatch and lower level of pigmentation). In addition, the chondrogenesis was deeply affected. As shown in Fig. 4A, the cartilage is not well defined in those progenies from treated sperm, the craniofacial skeleton formation being deeply affected. Immunodetection of the hallmarks of DNA repair (γH2AX and 53BP1) (Fig. 5) revealed that at the earliest stage (1k-cell) the embryos showed a much more intense repairing activity, particularly in batches from treated sperm. The presence of both γ-H2AX and 53BP1 (Fig. 5B,C) was twice as high in embryos from treated sperm than in those obtained from untreated sperm. A slight decrease in the repairing activity was observed at 8 hpf in the same batches when comparing to the control ones, but no differences were found at 24 hpf.

**Fig. 6.**
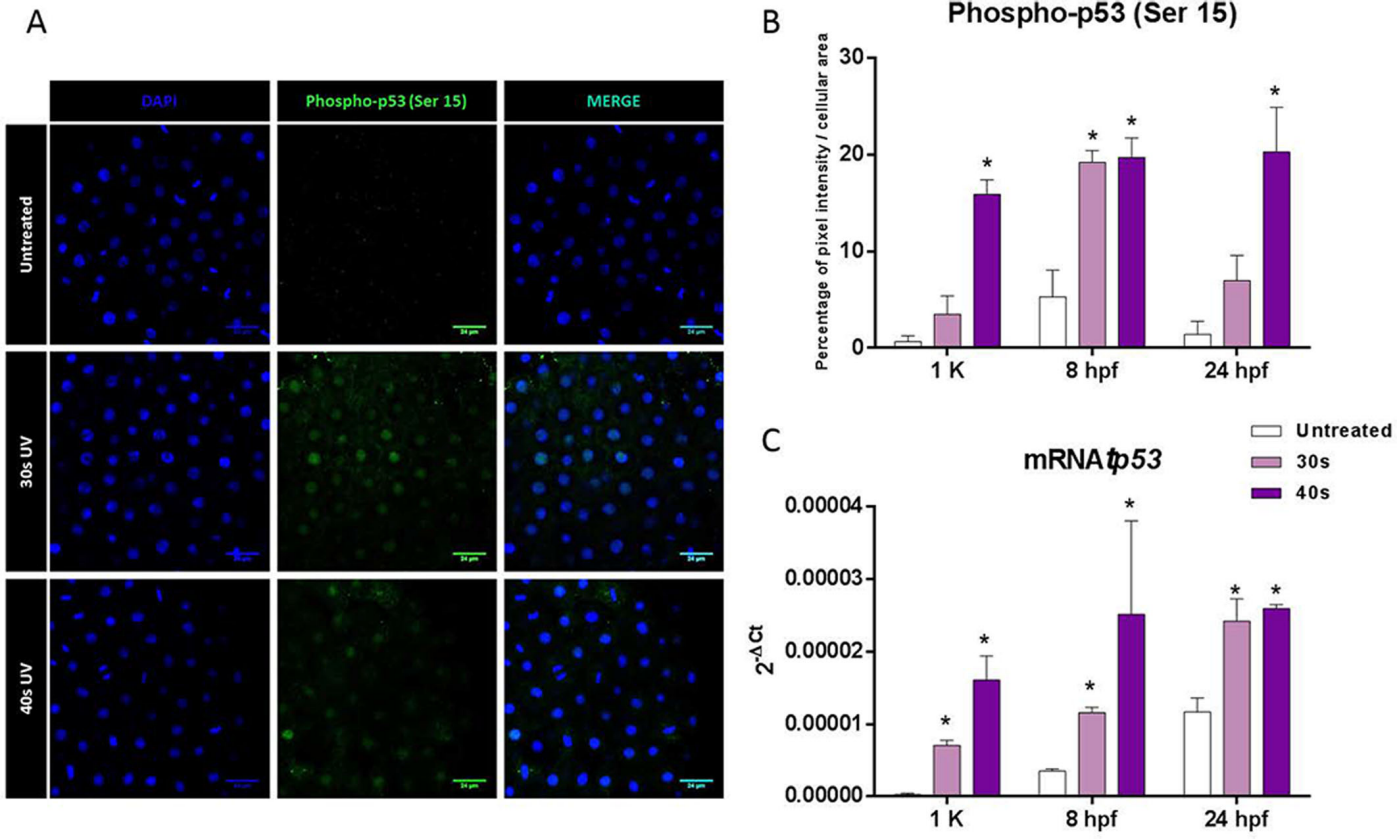
**The guardian of the genome: p53.** (A) Phopho-p53 (Ser 15) immunolocalization. Representative confocal images (40×) of whole embryos. Cell nuclei were stained with DAPI. Scale bars: 24 µm. (B) Percentage of pixel intensity of phospho-p53 (Ser 15) per cellular area. (C) mRNA levels of *tp53* at different stages during development (1k-cell, 8 hpf and 24 hpf) in progenies obtained from untreated sperm and sperm treated with 30 s and 40 s of UV irradiation. Data are mean±s.e.m. (*n*=3) (*P<0.05).

**Fig. 7.**
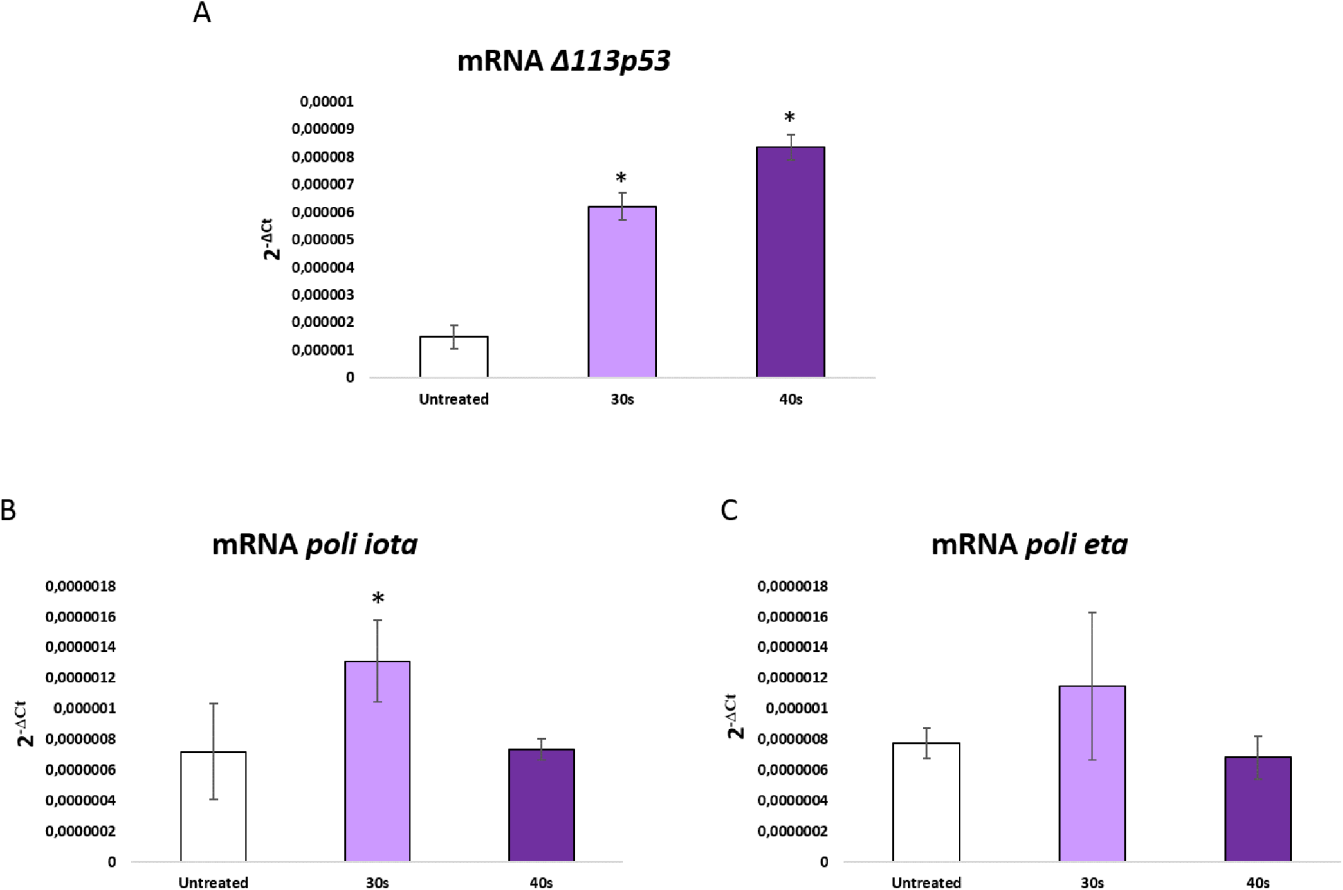
**mRNA levels of p53 isoform Δ*113p53* at 8 hpf in progenies from untreated and treated sperm with 30 s and 40 s of UV irradiation.** Data are mean±s.e.m. (*n*=3) (*P<0.05).

The immunodetection of p53-phosphorylated also indicated a very significant increase in the activation of p53 in the progenies from irradiated sperm for 40 s (Fig. 6A,B), particularly at 8 hpf. This pattern was consistent with the expression of *tp53* in the same developmental stage, showing an upregulation in those batches from DDS (Fig. 6C). Moreover, the alternative isoform Δ*113p53* was upregulated in the same batches (Fig. 7A). In the other hand, the analyzed polymerases did not show modified expression levels in the experimental progenies (Fig. 7B,C).

**Fig. 8.**
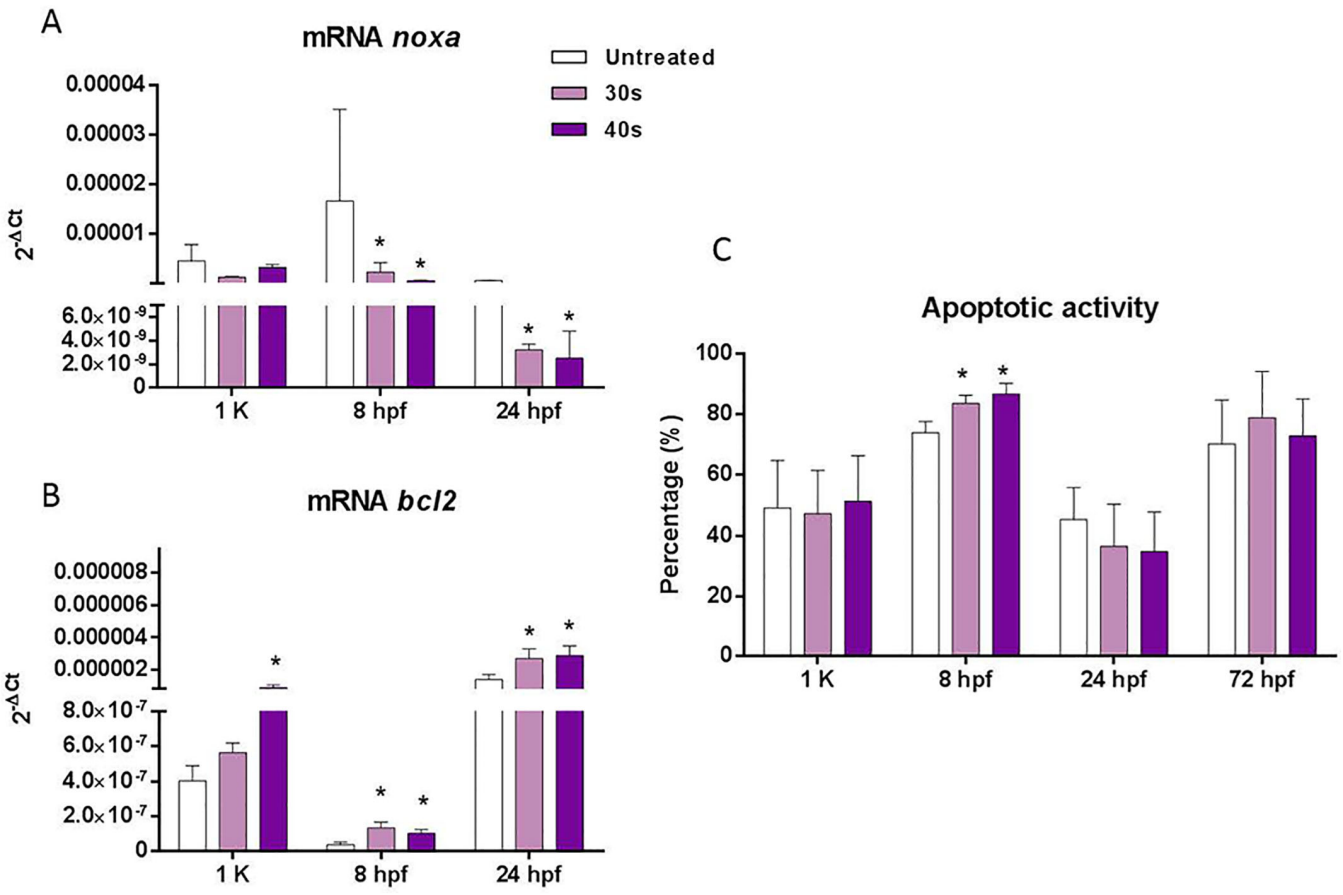
**Apoptotic downstream factors.** **(**A,B) mRNA levels of genes involved in the DNA damage response (DDR) during embryo development at 1k-cell, 8 hpf and 24 hpf in progenies from untreated sperm and sperm treated with 30 s and 40 s of UV irradiation: expression of *noxa* (A); expression of *bcl2* (B)*.* (C) Apoptotic activity measured by flow cytometry at different stages of development (1k-cell, 8 hpf, 24 hpf and 72 hpf). Data are mean±s.e.m. (*n*=3) (*P<0.05).

The expression of pro-apoptotic gene *noxa* (Fig. 8A) was significantly lower in embryos from irradiated sperm at 8 hpf and 24 hpf, but no changes were noticed during mid-blastula transition. *bcl2* showed an opposite pattern: a great increase in the expression of this gene was observed from zygotic activation (1k-cell) to organogenesis (24 hpf) in progenies from treated sperm (Fig. 8B). The apoptotic activity evaluated by the detection of annexin V varied during development, reaching the maximum at 75% epiboly (8 hpf). Progenies from treated sperm displayed a slightly enhanced apoptotic activity compared with those from control sperm at that stage (Fig. 8C).

## DISCUSSION

The good quality of the germinal cells is a mandatory condition to generate a healthy progeny. After the fusion of male and female gametes, a cascade of interactive events among oocyte and sperm components will determine the embryo performance (Anifandis et al., 2014). It is known that in mammals, DDS increase embryo genomic instability and lead to pregnancy loss, rise in number of abortions, birth defects and genetic diseases in the offspring (González-Marín et al., 2012; Marchetti et al., 2007, 2004; Robinson et al., 2012). The sperm used in this work were submitted to different levels of UV irradiation (30 s and 40 s) promoting severe lesions in all the treated cells. The genotoxic damage did not affect the fertilization ability, but severely compromised progeny development. It is known that the oocyte contains the transcripts and proteins in charge of repairing the sperm DNA lesions before the zygotic genomic activation (Jaroudi et al., 2009; Menezo et al., 2007). In most cases, the repairing ability compensates the spermatic damage and development progresses properly. However, the percentage of DNA lesions that can be repaired is limited (Gunes et al., 2015; Pérez-Cerezales et al., 2011). Some studies have revealed huge variations in the oocyte repairing ability (Fernández-Díez et al., 2016; Generoso, 1980). It is considered that trout oocytes are able to repair up to 10% of DNA damage (Pérez-Cerezales et al., 2011), Ahmadi and Ng (1999) estimated that less than 8% is repaired in mouse, whereas, according to Evenson and Jost (2000), human oocytes can repair up to 30% of DDS. The repairing capacity depends on the extent and type of DNA damage, as well as on the oocyte characteristics. The analyses performed in this work revealed that ∼80% of the sperm cells showed a range of fragmented DNA higher than 30% using comet assay, and that ∼20% of sperm cells were positive using TUNEL assay, very likely exceeding the normal capacity of zygotic repair. When DDS overcome the repairing ability, two scenarios can be generated: on the one hand, the damage is non-compatible with development driving to embryo death or, on the other hand, the development progresses in spite of having an unrepaired genome (González-Marín et al., 2012; Marchetti et al., 2004).

Early developmental stages are crucial as far as repairing capacity is concerned. It has been demonstrated that, in mammals, zygotic checkpoint senses DNA lesions activating the cell cycle arrest, the repairing pathways or even the apoptotic activity before the first mitosis, thus preventing chromosome fragmentation, embryo loss and infertility (Chen et al., 2012; Ladstatter and Tachibana-Konwalski, 2016; Marchetti et al., 2015; Wang et al., 2013). Some lesions are able to escape from this survival mechanism and suffer from defective repair, which results in chromosomal aberrations in the daughter cells (Marchetti et al., 2015). Marchetti et al. (2004) performed a cytogenetic analysis of the progenies from mice males treated with different mutagens, concluding that chromosomal aberrations paternally transmitted to the progeny were formed before the end of the first S phase after fertilization.

Our data revealed a significant genetic stress which required an intense repairing activity at the mid-blastula transition stage (MBT) (1k-cell) in those embryos obtained from treated sperm. At this stage, zebrafish embryos undergo the progressive transition from maternal to zygotic genome (Haberle et al., 2014; Lee et al., 2014). The high levels of γH2AX and 53BP1 indicate the recruitment of repairing factors at DSB sites (Durdik et al., 2015; Podhorecka et al., 2010), clearly suggesting an increased repairing effort in those embryos with DDS, which are more genetically instable. Nevertheless, we have not observed neither any delay in development nor any decrease in embryo viability, revealing that embryos go ahead with development despite the presence of a significant increased number of DNA-damaged spots. The dependence of the maternal genome (transcripts and proteins) at this stage could allow a normal development even in the presence of embryo genotoxic damage. It is also known that through mammalian development, death of embryos with structural aberrations does not seem to occur during the first cycles of development (Marchetti et al., 2004). This delay in development has been related to the control exercised by the maternal factors up to the full activation of the zygotic genome and to the inefficiency of the cell cycle checkpoints at early developmental stages.

At 75% epiboly (8 hpf), development progresses in all embryos, both from control and treated sperm, without showing signs of increased mortality rates. The DNA repair foci decreased at lower levels than in control batches, suggesting the end of the massive repairing activity of the paternal damage. An increase in apoptosis was observed at this stage, mainly in samples from DDS, which could reflect the activation of mechanisms avoiding the survival of cells with residual damage (Hakem et al., 2008), when the dependence on the embryonic transcripts is established (Haberle et al., 2014). This process represents a programmed cell death to facilitate a normal embryonic development or to prevent the spread of a localized lesion, allowing the survival of the organism as a whole (Montero et al., 2016).

Embryo death clearly increases at organogenesis (24 hpf), more intensively in embryos from DDS, but the DNA repairing effort and the apoptotic activity show the same levels than in control embryos. Approximately, less than half of the embryos from treated sperm were able to hatch when comparing to control batches and most of them showed severe malformations incompatible with long-term survival. The malformations affected different processes: chondrogenesis, skeleton morphogenesis, pigmentation, heart morphogenesis, angiogenesis and lymphatic vessels formation (or edemas), showing that organogenesis was severely impaired in embryos obtained with damaged sperm. The affected developmental processes are regulated by different pathways, indicating that development has progressed in spite of a misregulation of crucial processes in development. This fact indicates that the big repairing effort during the earlier stages was insufficient to properly repair the inherited DNA lesions. Moreover, it means that the survival mechanisms which face genotoxicity were not very restrictive during embryo development and were not activated in spite of such an intense instability.

The study of p53 expression and activation (amplification of *p53* transcripts by RT-PCR and immunodetection of p53P) showed a clear activation of the tumor suppressor factor p53 in the progenies from DDS at all the developmental stages, both at transcriptional and post-translational levels. This activation is usually followed by the downstream activation of the surveillance mechanisms against genotoxic damage. The downstream events are mediated by the transcriptional activity of p53, which could affect a wide number of genes driving to cell cycle arrest, to DNA repair or to apoptosis (Vousden and Lu, 2002). Our results showed that development progresses without signs of an increased apoptotic activity. Moreover, regardless of the high expression and activation of p53, the downstream pro-apoptotic factor *noxa* showed a significant downregulation, whereas the anti-apoptotic gene *bcl2* was upregulated. The balance between pro- and anti-apoptotic genes suggests a repressive apoptotic scenario in batches from DDS that would repress the loss of cells with unrepaired damage.

The activation of p53 in absence of an enhanced apoptosis or repairing activity could point to the potential implication of p53 in other pathways or to its downstream inhibition. In fact, a role of p53 as an enhancer of cell tolerance to DNA damage has been recently described in UV irradiated cells (Hampp et al., 2016; Lerner et al., 2017). DNA unrepaired lesions may evade detection and persist into S-phase. Such a process is referred as DDT or lesion bypass. DNA lesions usually block the advance of polymerases at replicative forks, but, from a cell survival perspective, bypassing of stalled forks can be accomplished by specific DNA polymerases (POL eta, POLi), dependent on the activation of p53 (Hampp et al., 2016; Lerner et al., 2017). Thus, the direct role of p53 in the translesion DNA synthesis has a protective effect on cell survival and a key role in DDT. However, the expression of *poli eta* and *iota* polymerases was not modified in the experimental progenies, suggesting that other mechanisms would mediate the implication of p53 in DNA damage tolerance. In fact, a different mechanism has been identified in zebrafish embryos: an alternative isoform of p53 (*Δ113p53*), initiated from an alternative promoter which generates a shorter transcript, is transactivated by the canonical p53 in response to developmental and DNA damage signals. This short-form of tp53 antagonizes the p53-depending apoptosis through the activation of the anti-apoptotic factor *bcl2* (Chen et al., 2009). Therefore, the expression of *Δ113p53* generates a feedback pathway that modulates the p53 response, promoting cell survival under specific circumstances. Our results clearly revealed that the instability generated by paternal DNA damage promotes the expression of Δ*113p53*, likely mediating the high level of tolerance to DNA damage up to hatching in spite of an abnormal development.

Downregulated apoptosis was also observed in embryos from other external fertilizers submitted to different kind of genotoxic stress: corals in an acidic environment (Tchernov et al., 2011), trout embryos for which zygotic DNA repair activity was inhibited (Fernández-Díez et al., 2015) and trout embryos from oocytes with a compromised quality (Fernández-Díez et al., 2016). The variable efficiency of DNA signalization and repairing pathways, during gametogenesis and embryo development, tolerates a limited number of mutations, allowing the evolution to take place (Jaroudi and Sengupta, 2007). A higher level of tolerance in fish embryos than in mammals could lie behind the survival mechanism of animals with a reproductive strategy based on the production of a great number of siblings with a low rate of long-term survival. Tolerance to unrepaired DNA from sperm could introduce new mutations, some of them potentially advantageous to face a changing environment. The enhanced expression and activity of p53 together with the repressed apoptotic activity point to the activation of p53 dependent DDT pathways, specifically the transactivation of Δ*113p53*, as the mechanism responsible for such genotoxic tolerance that deserves a deeper study.

## MATERIALS AND METHODS

### Reagents

All media components were purchased from Sigma-Aldrich except when otherwise stated.

### Ethics statement

The experiments were carried out in accordance with the Guidelines of the European Union Council (86/609/EU, modified by 2010/62/EU), following Spanish regulations (RD 1201/2005, abrogated by RD 53/2013) for the use of laboratory animals, and were approved by the Ethics and Scientific Committee of the University of Leon and the Competent Organism of the Junta de Castilla y León (project number ULE008-2016).

### Collection of gametes and *in vitro* fertilization

Mature eggs and sperm were obtained by gentle squeezing of males and females following the procedures by Hagedorn and Carter (2011). Animals were immersed in a solution of tricaine methane sulfonate (MS-222) according to Westerfield (2002) until gill movements had slowed. Males were placed in a sponge with the ventral surface up. The anal fin area was dried and both sides from pectoral to the anal fin were pressed using forceps. Sperm were collected with a 10 µl pipette and then placed into a 1.5 ml plastic tube (Eppendorf) containing 20 µl PBS on ice. The same tube was used to pool the sperm from five males. Pooled sperm was divided into three aliquots, each one containing ∼1×10^7^ cell/ml. One aliquot was used as a control, and the rest were subjected to different doses of UV-C irradiation.

To collect the eggs, anesthetized females were dried and placed in a Petri dish. Pressure was applied with a slight movement of the fingers on the ventral side, back towards the pelvic fins. The eggs were immediately fertilized using 50 µl diluted pooled sperm (from control or treated samples) and incubated for 2 min with 750 µl water to activate the gametes.

### Sperm UV irradiation

Each cell suspension was transferred to a 35-mm plastic Petri dish and placed on ice at 15 cm under a UV lamp (Vilber Lourmat, Eberhardzell, Germany) (254 nm), receiving 400 CW/cm^2^ irradiation for 30 s or 40 s – time enough to promote a high rate of DNA fragmentation without affecting fertilization ability and allowing survival up to hatching stage, as was demonstrated in a preliminary study irradiating for 10, 20, 30, 40 and 50 s (Table S1). Then, control and treated samples were kept in the dark at 4°C until further analysis.

### Analysis of sperm chromatin integrity

#### Comet assay

DNA fragmentation was analyzed using the Single Cell Gel Electrophoresis (SCGE) or Comet assay. Control and treated samples were diluted in PBS 1× to a final concentration of 1×10^6^ cell/ml. Samples treated with 20 µM H_2_O_2_ during 15 min on ice were used as a positive control. All samples were centrifuged at 8000× ***g*** for 5 min at 4°C, and the pellet was re-suspended in 10 µl PBS 1×. Sperm cells were mixed with 180 µl of 0.5% low melting point agarose (Promega Biotech Iberica, Madrid, Spain) and 75 µl of cell suspension were pipetted over a 3-Aminopropyl-triethoxysilane (ATE)-treated slide and covered with a glass cover slip. Slides with gels were kept at 4°C for 30 min to solidify. Then, the coverslips were removed and the slides were incubated 1 h at 4°C in lysis solution (100 mM EDTA-Na_2_, 2.5 M NaCl, 10 mM Tris-HCl and 1% Triton X-100, pH 10). The slides were placed into electrophoresis buffer (1 mM EDTA-Na_2_, 0.3 M NaOH, pH 13) for 20 min to reach DNA unwind, followed by 20 min of electrophoresis (25 V, 280-350 mA). The slides were washed using a neutralization solution (0.4 M Tris-HCl, pH 7.5). This procedure was repeated three times to ensure the elimination of all alkali and detergents. The slides were then fixed with absolute methanol for 3 min and left to dry, always being protected from the light.

Twenty microliters of 0.5 µg/ml 4′, 6-diamidino-2-phenylindole (DAPI) were pipetted over the slides for comet visualization and were covered with a coverslip. Samples were observed with an epifluorescence microscope (Eclipse E800, Nikon, Tokyo, Japan) fitted with a 510-560 nm excitation filter and a 590 nm barrier filter. Images were obtained with a Nikon DXM1200F digital camera, acquiring ∼50 cells from each slide using ACT-1 software (v. 2.62, Nikon). Images were analyzed with the free CaspLab software (1.2.3beta2; http://www.casp.of.pl.) and the percentage of tail DNA (% DNAt) was used to quantify the DNA damage.

#### TUNEL assay

A terminal deoxynucleotidyl transferase dUTP nick end labelling (TUNEL) kit (Roche, Penzberg, Germany) was used to detect DNA fragmentation. Control and treated (30 s and 40 s UV irradiation) samples were washed two times with PBS 1× and then fixed with 4% paraformaldehyde in PBS 1× for 20 min at room temperature (RT). Samples were centrifuged at 8000× ***g*** at 4°C for 5 min and the pellet was re-suspended with 100 µl distilled water. Twenty microliters of the cell suspension were dropped over each slide. The slides were dried at 37°C overnight (ON).

The samples were permeabilized with 0.1% Triton X-100 in 0.1% sodium citrate for 5 min at RT and washed in PBS 1×. Thirty microliters of TUNEL reaction mixture were added per drop, and the slides were kept in a wet chamber for 1 h at 37°C in darkness. A negative staining was performed as control using 30 µl of label solution without enzyme solution. The positive control of fragmentation samples was treated for 10 min at 25°C with 30 µl of a solution containing 1 µl TURBO DNase (Applied Biosystems, Foster City, USA), 1 µl TURBO DNase (Applied Biosystems, USA) buffer 10× and 26 µl of distilled water, before performing TUNEL reaction.

Slides were washed twice in PBS 1× and were labelled with 50 µM DAPI for 5 min at RT. The slides were washed three times and were mounted with ProLong Gold Antifade reagent (Thermo Fisher Scientific), covered with a coverslip and eventually analyzed using a confocal microscope LSM 800 (Axio Observer Z1, Zeiss, Oberkochen, Germany).

### Analysis of the progeny performance

#### Fertilization rates and embryo development

Embryos were kept at 28°C in darkness until hatching. Fertility rates at 1k-cell, accumulative mortality rates throughout embryo development at 1k-cell, 8 hpf, 24 hpf, 48 hpf and 72 hpf, and survival rates after hatching (72 hpf) were evaluated.

#### Malformations

At 72 hpf, the percentage of malformed embryos was analyzed. Those embryos unable to naturally hatch were manually dechorionized in order to characterize the developmental abnormalities. Malformations were classified in eight types: axial torsion, distal torsion, defective yolk-sac, cardiac edema, no hatching, no caudal fin, no pectoral fin and defective pigmentation. To characterize the head skeletal malformations, the cartilage was stained in larvae at 6 days post-hatching (dph). Ten larvae from each batch were fixed using 4% paraformaldehyde solution at 4°C ON. The next day, larvae were washed three times with tap water and then with PBT solution (0.1% Tween-20 in PBS 1×). Then, embryos were cleared using 1.5% H_2_O_2_ in1% KOH during 30 min and washed twice in PBT. The whole larvae were stained with 0.1% Alcian Blue in 70% EtOH. After staining, they were washed with 70% EtOH in 5% HCl and 0.25% KOH in 20% glycerol for 3 h each wash. Finally, they were kept in 0.25% KOH in 50% glycerol ON and the next day were stored at 4°C in 0.1% KOH in 50% glycerol to acquire the images using a stereomicroscope.

### DNA damage response (DDR)

#### DNA repairing activity: γH2AX and 53BP1 immunodetection

Ten alive embryos from batches derived from control and treated sperm at 1k-cell, 8 hpf and 24 hpf were fixed with 4% paraformaldehyde in PBS 1× ON at 4°C. The embryos were washed three times with tap water, and then were de-chorionized and permeabilized with pure methanol for 2 h at −20°C. Then, they were incubated with 2 N HCl for 1 h at RT to denature the DNA. The embryos were washed twice with PDT [1× PBST (0.1% Tween 20 in PBS 1×), 0.3% Triton and 1% DMSO] for 20 min at RT. After that, they were incubated in blocking solution (20% goat serum, 3% BSA in PBST) for 1 h at RT and transferred to blocking solution with diluted primary antibodies: mouse monoclonal to γ-H2AX (phospho S139) (ab 26350, 1:100 at the two earlier stages and 1:50 at 24 hpf) and rabbit polyclonal to 53BP1 (ab36823, 1:200 at the two first stages and 1:100 at 24 hpf) for 2 days at 4°C. Embryos were washed with PDT solution and then incubated for 1 day at 4°C with secondary antibodies (A865 and A11008, both from Thermo Fisher Scientific) in blocking solution.

All samples were washed twice and labelled with 50 µM DAPI for 10 min at RT. The slides were washed, mounted with ProLong Gold Antifade reagent and covered with a coverslip. The samples were observed with a confocal microscope LSM 800 (Observer Z1, Zeiss). Nuclear fluorescence emission of 300 randomly selected cells per embryo (five embryos per treatment) was quantified using ImageJ software (https://imagej.nih.gov/ij/).

#### tp53 activation analysis

The immunodetection of p53 phosphorylated [Phospho-p53 (Ser15) Antibody from Cell Signaling Technology] was performed as previously described for DNA repairing activity. The primary antibody (1:50) was incubated 2 days at 4°C in blocking buffer in embryos at 1k-cell, 8 hpf and 24 hpf. The secondary antibody (A11008, Thermo Fisher Scientific) was incubated for 1 day at 4°C in blocking buffer. The samples were analyzed in triplicate, and were observed with a confocal microscope LSM 800 (Observer Z1, Zeiss). Nuclear fluorescence emission in 300 randomly selected cells per embryo (five embryos per treatment) were quantified using ImageJ software.

#### Apoptotic activity

Apoptotic activity was analyzed using cells pooled from 10 alive embryos at different developmental stages (1k-cell, 8 hpf, 24 hpf and 72 hpf). Embryos at 8 hpf and 24 hpf and larvae at 72 hpf were dechorionized and cut in small pieces. The fragments were incubated for 2 h under agitation in a dissociation solution containing 6 mg/ml collagenase I, 2.4 µl DNAse I (Applied Biosystems, USA), and 10% (v/v) FBS in Leibovitz's (L-15) medium. The 1k-cell embryos were also dechorionized and repeatedly pipetted in L-15 medium to promote the dissociation process. Then, cells were filtered using a 100-mm nylon mesh and washed twice with L-15 medium. Apoptotic activity was detected using a FITC annexin V Apoptosis Detection Kit (Molecular Probes by Life Technologies) following the manufacturer's instructions. The samples were analyzed with an ImageStream multispectral imaging flow cytometer (Amnis Corporation, Seattle, USA) using a 488 nm laser, and data analysis was performed using IDEAS software [IDEAS: Image Data Exploration and Analysis Software (6.1 V)].

### Expression of genes related to DDR

#### RNA extraction and reverse transcription

Total RNA from embryos at 1k-cell, 8 hpf and 24 hpf, and from larvae at 72 hpf, was obtained using a Trizol^®^ Reagent kit (Applied Biosystems, Madrid, Spain) following the manufacturer's instructions. RNA integrity was confirmed by electrophoretic analysis of total RNA samples prior to reverse transcription (data not shown). Total RNA concentration was determined using a NanoDrop ND-1000 Spectrophotometer (Thermo Fisher Scientific). One microgram of total RNA from embryos and larvae were reverse transcribed using the High Capacity cDNA Kit (Applied Biosystems, Spain) following the manufacturer's instructions.

#### Gene expression

Reverse-transcribed products from the extracted RNA were used to perform a quantitative polymerase chain reaction (qPCR) assay to analyze the expression of genes involved in DNA damage response*: noxa* (pro-apoptotic gene), *bcl2* (anti-apoptotic gene), *tp53* (considered as ‘genome guardian’), a *p53* isoform, Δ*113p53* and two polymerases: *poli eta* and *poli iota*. 600 ng of cDNA products were used for each qPCR. The primers were designed using Primer Express Software v2.0 (Applied Biosystems, Spain) and Primer Select Software v10.1 (DNASTAR, Lasergene Core Suite; https://www.dnastar.com/t-help-primerselect.aspx). The primer sequences and accession number are summarized in Table 1. Product specificity was checked by melting curves, and product size was visualized by electrophoresis on agarose gel (data not shown). Reaction mixtures (total volume 20 µl) contained 600 ng of cDNA, 10 µl of 1× SYBR Green Master mix (Applied Biosystems, Spain) and 0.5 µl of 10 µM each forward and reverse primer. qPCR was initiated with a pre-incubation phase of 10 min at 95°C followed by 40 cycles of 95°C denaturation for 15 s, annealing for 1 min at the optimal temperature for each pair of primers. Three technical replicates were performed per sample. Expression level for each repairing gene was normalized to 18S RNA gene using the Delta-Ct (2ΔCt) method to analyze relative changes in gene expression concerning the housekeeping expression.

**Table 1.**
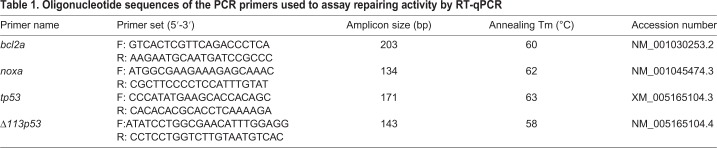
**Oligonucleotide sequences of the PCR primers used to assay repairing activity by RT-qPCR**

### Statistical analysis

Data analysis was carried out using a computerized package generated by SPSS 24.0 software for Windows (IBM, EEUU). The results were expressed as mean±s.e.m. One-way ANOVA test followed by DMS or Games Howell post hoc test (*P*<0.05) was used to analyze parametric data.

## Supplementary Material

Supplementary information
