# Supplementary material for: Tolerance to paternal genotoxic damage promotes survival during embryo development in zebrafish (Danio rerio)
Source: Biol Open. 2018 May 15;7(5):bio030130. doi: 10.1242/bio.030130 (PMC5992526; doi:10.1242/bio.030130)
Supplement: Supplementary information [file biolopen-7-030130-s1.pdf]

Supplementary table 1: Fertility and hatching rates of embryos fertilized with sperm submitted at different doses of UV irradiation in a preliminary study.

Time UV irradiation	Fertility rate (%)	Hatching rate (%)
10s	94,2	31,8
20s	96,8	25,7
30s	93,9	20,6
40s	92,1	15,4
50s	77,6	1,02

Biology Open • Supplementary information
